# The Molecular Chaperone Binding Protein BiP Prevents Leaf Dehydration-Induced Cellular Homeostasis Disruption

**DOI:** 10.1371/journal.pone.0086661

**Published:** 2014-01-29

**Authors:** Humberto H. Carvalho, Otávio J. B. Brustolini, Maiana R. Pimenta, Giselle C. Mendes, Bianca C. Gouveia, Priscila A. Silva, José Cleydson F. Silva, Clenilso S. Mota, Juliana R. L. Soares-Ramos, Elizabeth P. B. Fontes

**Affiliations:** 1 National Institute of Science and Technology in Plant-Pest Interactions, Universidade Federal de Viçosa, Viçosa, MG, Brazil; 2 Departamento de Bioquímica e Biologia Molecular/Bioagro, Universidade Federal de Viçosa, Viçosa, MG, Brazil; 3 Departamento de Biologia Vegetal, Universidade Federal de Viçosa, Viçosa, MG, Brazil; RIKEN Center for Sustainable Resource Science, Japan

## Abstract

BiP overexpression improves leaf water relations during droughts and delays drought-induced leaf senescence. However, whether BiP controls cellular homeostasis under drought conditions or simply delays dehydration-induced leaf senescence as the primary cause for water stress tolerance remains to be determined. To address this issue, we examined the drought-induced transcriptomes of BiP-overexpressing lines and wild-type (WT) lines under similar leaf water potential (ψ_w_) values. In the WT leaves, a ψ_w_ reduction of −1.0 resulted in 1339 up-regulated and 2710 down-regulated genes; in the BiP-overexpressing line 35S::BiP-4, only 334 and 420 genes were induced and repressed, respectively, at a similar leaf ψ_w_ = −1.0 MPa. This level of leaf dehydration was low enough to induce a repertory of typical drought-responsive genes in WT leaves but not in 35S::BiP-4 dehydrated leaves. The responders included hormone-related genes, functional and regulatory genes involved in drought protection and senescence-associated genes. The number of differentially expressed genes in the 35S::BiP-4 line approached the wild type number at a leaf ψ_w_ = −1.6 MPa. However, N-rich protein (NRP)- mediated cell death signaling genes and unfolded protein response (UPR) genes were induced to a much lower extent in the 35S::BiP-4 line than in the WT even at ψ_w_ = −1.6 MPa. The heatmaps for UPR, ERAD (ER-associated degradation protein system), drought-responsive and cell death-associated genes revealed that the leaf transcriptome of 35S::BiP-4 at ψ_w_ = −1.0 MPa clustered together with the transcriptome of well-watered leaves and they diverged considerably from the drought-induced transcriptome of the WT (ψ_w_ = −1.0, −1.7 and −2.0 MPa) and 35S::BiP-4 leaves at ψ_w_ = −1.6 MPa. Taken together, our data revealed that BiP-overexpressing lines requires a much higher level of stress (ψ_w_ = −1.6 MPa) to respond to drought than that of WT (ψ_w_ = −1.0). Therefore, BiP overexpression maintains cellular homeostasis under water stress conditions and thus ameliorates endogenous osmotic stress.

## Introduction

The endoplasmic reticulum (ER) is a major biosynthetic organelle in all eukaryotic cells and it is the center secretory proteins synthesis, which occurs in ER membrane-associated polysomes, and protein processing, which occurs in the luminal space. The ER quality control (ER-QC) system mediates and monitors secretory proteins processing and folding, identifies and presents misfolded proteins to the ER-associated degradation (ERAD) machinery and thereby ensures that only properly folded proteins proceed to their final destinations in the secretory pathway [1]. To perform this task, ER-QC relies on molecular chaperone activities that not only assist in proper folding but also monitor the unfolded status of the secretory proteins. One such ER-resident molecular chaperone is the binding protein (BiP), which has been demonstrated to have multiple functions. BiP mediates the folding and maturation of secretory proteins, the targeting of misfolded proteins for degradation, the translocation of secretory proteins into the ER lumen and the regulation of the unfolded protein response (UPR), a signaling cascade that allows the ER lumen to communicate with the nucleus and cytoplasm of ER-stressed cells [2]–[4].

As in other eukaryotes, plant BiP is induced by any conditions that disrupt ER homeostasis and cause unfolded protein accumulation in the lumen of the organelle, a condition known as ER stress [5], [6]. To cope with the effects of ER stress, the cytoprotective UPR is activated and ER-resident molecular chaperone-, ERAD- and secretory route component-encoding genes are induced to increase the ER protein folding and processing capacity under stress conditions [2], [7], [8]. However, under persistent stress conditions resulting in an insufficient protein-processing capacity, the unfolded proteins are translocated back to the cytosol to be degraded by the proteasome through the activation of the ERAD system [9]–[12]. BiP is directly involved in ER- associated protein degradation by recognizing and targeting abnormally folded proteins for degradation [13]

The UPR can be activated by a variety of biotic and abiotic stresses that cause the accumulation of unfolded proteins in the ER lumen [14], [15]. Furthermore, chemical agents, such as tunicamycin and dithiothreitol (DTT), which interfere with protein folding in the ER, can induce the UPR [11]. In mammalian cells, the UPR is transduced through three distinct ER transmembrane sensors, namely PERK (protein kinase RNA-like ER kinase), Ire1 (inositol-requiring enzyme-1) and the basic leucine zipper transcription factor ATF6 (activating transcription factor-6) [16]. The molecular chaperone BiP has been shown to play a pivotal role in controlling the activation status of IRE1, PERK and ATAF6 [17]. Under normal conditions, BiP binding to the luminal domain of these UPR transducers renders them inactive, whereas the release of BiP from these sensors, which is triggered by unfolded protein accumulation in the ER, signals the activation of UPR.

Upon the disruption of ER homeostasis, plant cells activate at least two branches of the UPR through IRE1-like and ATF6-like transducers, resulting in the up-regulation of ER-resident molecular chaperones and the activation of the ER-associated protein degradation system [18]. The plant Ire1 ortholog is represented by two copies in the Arabidopsis genome, namely Ire1a and Ire1b, and one copy of OsIre1, in the rice genome [19], [20]. Similar to its mammalian counterpart, plant Ire1 is associated with the ER membrane and exhibits ribonuclease and autophosphorylation activities [21]. The substrate for plant Ire1 endonuclease activity is the transcript of the ER membrane-associated transcription factor bZIP60. In response to ER stress, bZIP60 mRNA is spliced in an IRE1-mediated process to generate an alternatively spliced transcript that lacks transmembrane domain-encoding sequences [21], [22]. This splicing leads to the synthesis of a soluble and functional bZIP60 transfactor that can be translocated to the nucleus, where it activates ER stress-inducible promoters. In addition, rice OsbZIP74 or OsbZIP50, which is an ortholog of Arabidopsis AtbZIP60, is regulated through the IRE1-mediated splicing of its RNA. In maize *ZmbZIP60* transcript splicing leads to the activation of ER stress-inducible promoters [23]–[25]. The second branch of UPR in plants mechanistically resembles the ATF6-mediated transduction of the ER stress signal. In responding to ER stress, the membrane-associated Arabidopsis ATF6 homologs bZIP17 and bZIP28 are relocated to the Golgi, where their transcriptional domains are proteolytically released from the membrane by SP2 [26], [27]. The released bZIP domain of these transfactors is then translocated to the nucleus, where it acts in concert with the heterotrimeric NF-Y complex to activate UPR genes [14]. Plant BiP has also been implicated in the regulation of UPR. BiP overexpression in tobacco and soybeans attenuates the activation of the UPR [28], [29] and, in Arabidopsis, BiP was recently shown to bind to and regulate bZIP28 activity [30]. Under unstressed conditions, plant BiP is bound to a bZIP28 that remains in the ER membrane. In response to ER stress, BiP dissociates from bZIP28, allowing it to be mobilized from the ER to the Golgi where it is proteolytically processed and released to enter the nucleus. Plant BiP has also been shown to modulate a stress-induced N-rich protein (NRP)-mediated cell death response [31]–[33].

NRP-mediated cell death signaling is a distinct, plant-specific branch of the ER stress pathway that has been uncovered in soybeans and has been shown to integrate ER and osmotic stress signals into a full response [34]. Ever since the discovery of the stress-induced NRP-mediated cell death response, many aspects of this signaling pathway induced by prolonged ER and osmotic stress have been detailed. We now know that both NRP-A and NRP-B in soybean, which harbor a conserved development and cell death domain (DCD) at the N-termini, are critical mediators of a stress-induced cell death response, which in turn resembles a programmed cell death event [28]. NRP expression is controlled by the ER and osmotic-stress induced transcriptional factor GmERD15, which specifically binds to the NRP-A and NRP-B promoters to activate their transcription [35]. Enhanced NRP accumulation causes the induction of plant-specific transcriptional factors GmNAC81 and GmNAC30, which interact with each other to fully activate the expression of the vacuolar processing enzyme (VPE), a plant-specific executioner of programmed cell death that displays caspase-1 like activity [36].

Because it is the sole molecular chaperone involved in the activation of UPR, BiP expression changes could indicate alterations in the folding environment and ER processing capacity. Accordingly, BiP overexpression in mammals and plants attenuates ER stress and suppresses UPR activation [29], [38]. In addition to alleviating ER stress [29], BiP overexpression in plants has also been shown to increase water deficit tolerance [33], [39], [40]. BiP-mediated increases in water stress tolerance have been associated with the capacity to delay drought-induced leaf senescence, which is mediated by NRP-mediated cell death signaling. In transgenic lines, BiP overexpression attenuates ER and osmotic stress-induced cell death phenotypes, such as foliar necrotic lesions and wilting, the percentage of dead cells, senescence-associated gene marker induction, DNA fragmentation, caspase activity, lipid peroxidation and the induction of cell death marker genes NRP-A, NRP-B and GmNAC6 [31]. These results implicate BiP as a negative regulator of the stress-induced NRP-mediated cell death response. Nevertheless, whether BiP controls cellular homeostasis under drought conditions or simply delays dehydration-induced leaf senescence as the underlying mechanism for BiP-mediated increase in drought tolerance remains to be determined. To address this issue, we compared the drought-induced transcriptomes of wild type (WT) soybean leaves and BiP-overexpressing leaves at a similar leaf water potential (ψ_w_), which was induced by a slow soil-drying regime. Our results revealed that a gradual reduction of the leaf ψ_w_ to −1.0 MPa imposed a sufficiently high level of dehydration stress to induce tremendous reprogramming of the gene expression in WT soybean leaves. In contrast, the BiP-overexpressing leaves maintained cellular homeostasis at ψ_w_ = −1.0 MPa because they displayed only a slight change in the expression profile compared to the irrigated, well-watered leaves. However, as the dehydration stress increased to a ψ_w_ = −1.6 or −1.7 MPa, the global gene expression variation in BiP-overexpressing lines and WT lines approached similar levels, although the expression of senescence-associated genes and UPR markers remained lower in BiP-overexpressing lines.

## Materials and Methods

### Plant growth

The transgenic lines 35S::BiP-2 and 35S::BiP-4 were previously obtained by transforming soybean plants (*Glycine max* cv. Conquista) with a *soy*BiPD gene (GeneBank accession AF031241) under the control of a *cauliflower mosaic virus* 35S promoter [33], [41]. Soybean seeds from untransformed, WT lines and transgenic lines, T5 generation, were germinated in organic substrate, transferred to 3-L pots containing a mixture of soil, sand and dung (3∶1∶1) and grown in a greenhouse under ambient light, relative humidity (65–85%) and temperature (15–35°C) conditions. Each pot was weighed and received an equal amount of soil mixture.

### Water stress induction in 35S::BiP-4, 35S::BiP-2 and WT lines in the greenhouse

Plants were grown under normal conditions and a normal water supply until reaching the V3 developmental stage (fully expanded third trifoliate) when the field capacity was measured in all pots. Control plants were watered daily with approximately 180 mL of water per plant. The transgenic lines 35S::BiP-4 and 35S::BiP-2 and the WT lines were exposed to a slow drying soil treatment that consisted of a reduction in irrigation to 40% of the daily normal water supply for 25 d [33]. At the V3/V4 developmental stage, one-half of the transgenic and WT plants received 40% of the normal irrigation (restricted water regime) for 25 d and the remaining 50% of plants received a continuous normal water supply (control).

The soil water content of all samples was recorded as a function of time to ensure that the extent of soil drying or the severity of the plant water stress was similar for all analyzed samples. The leaf water potential (ψ) was measured on the third emergent trifoliate at dawn by using a Scholander pump [42] during the stress period. The stress severity was also determined by measuring leaf relative water content (RWC). As BiP-overexpressing and WT leaves displayed a similar degree of leaf osmotic adjustment under the water deprivation regime [33], the decrease in the leaf ψ and RWC occurred at similar extent in 35S::BiP-4 and WT leaves. For example, a decline of the leaf ψ to −1.5 MPa was associated with RWC = 55.5±1% (n = 3) in WT leaves and RWC = 56±1% (n = 3) in 35S::BiP-4 leaves, under our experimental conditions, The leaf RWC of well-watered WT and 35S::BiP-4 leaves was approximately 87%.

### Total RNA isolation and mRNA purification

Total RNA was isolated from fully expanded third trifoliates using TRIzol and then treated with RNase-free DNase (*Life Technologies Inc*). The RNA was quantified by spectrophotometry (*Evolution 60 Thermo Scientific*) and the quality and integrity of the RNA was examined by using an ethidium bromide-stained agarose gel (1.3% p/v; 2% formaldehyde). PolyA+ was purified by using the FastTrack 2.0 mRNA Isolation Kit (Life Technologies Inc.) according to the instructions from the manufacturer, quantified by spectrometry (Evolution 60 Thermo Scientific) and examined by electrophoresis.

### Microarray experiments and data analysis

For the microarray, RNA was extracted from well-watered and drought-stressed untransformed, wild-type and BiP-overexpressing soybean leaves at the V3/V4 developmental stage. Three biological replicates corresponding to transformed (35S::BiP) and untransformed (WT) leaves were used to obtain two independent mRNA pools to hybridize duplicate chips for each sample. Hybridizations were performed on Affymetrix GeneChip Soybean Genome Arrays (http://affymetrix.com/index.affx) according to the manufacturer's instructions and by following these steps: (a) double-stranded cDNA synthesis with the SuperScript *One-Cycle cDNA Kit*, (b) purification and cleaning of double-stranded cDNA with the cDNA Wash Buffer and Cleanup Spin Column, c) synthesis of biotinylated cRNA using the IVT Labeling kit, (d) amplification of cRNA using the IVT Master Mix, purification as in b and quantification at 260 nm by spectrophotometry (Evolution 60 Thermo Scientific) and (e) fragmentation (hydrolysis) of biotinylated cRNA to 35 to 200 nucleotides. Then, the cRNA (15 µg) was hybridized to an Affymetrix Soybean Genome Array for 16 h according to the Affymetrix GeneChip protocol with buffers provided by the manufacturers. The arrays were washed in a GeneChip Fluidic Station 450 GCOS/Microarray Suite (GeneChip Operating Software) and stained using a GeneChip IVT Labeling Kit. Chips were scanned with a Gene Array Scanner 3000 (Affymetrix) at a 3 µm pixel resolution and a 570 nm wavelength to generate cell intensity files (CEL). The CEL files were computationally analyzed using the R/Bioconductor package (Version 1.6/Version 2.6.0) [43]. Normalization was performed using the Robust Multiarray Analysis (RMA) method [44], which is available through the R/Bioconductor package for Affymetyrix arrays [45]. A Microarray Quality Assessment was performed by using functions from the Affy package along with functions from the R/Bioconductor package. To identify differentially expressed genes, we used R/MAANOVA (Microarray Analysis of Variance, http://churchill.jax.org/software/rmaanova.shtml), and the significance of differential gene expression was detected with F and t tests. The estimated p-values were corrected by FDR (false discovery rate) [46], and the resulting gene lists were classified according to the corrected p-values and fold-changes. The genes were considered to be differentially expressed if they yielded a corrected p-value<0.05 and a fold-change >1.5. The genes were annotated using the annotation Affymetrix file for the chip (http://www.affymetrix.com/support/technical/annotationfilesmain.affx), which was complemented with annotations from the Soybase (http://www.soybase.org/) and Phytozome (http://www.phytozome.net/) databases. The microarray data was deposited at Gene Expression Omnibus database under the accession number GSE50408.

### Real time PCR

For quantitative RT-PCR, total RNA was extracted from the frozen leaves with TRIzol and treated with RNase-free DNase I. First-strand cDNA was synthesized from 3 µg of total RNA using 5 µM oligo-dT(18), 0.5 mM dNTPs and 1 U M-MLV Reverse Transcriptase (Life Technologies Inc.). All of the real-time PCR procedures, including tests, validations and experiments, were conducted according to the recommendations of Applied Biosystems. RT-PCR reactions were performed on an ABI7500 instrument (Applied Biosystems) using cDNAs from the treatments, gene-specific primers (Table S1) and the SYBR Green PCR Master Mix (Applied Biosystems). Amplification reactions were performed as follows: 10 min at 95°C and 40 cycles of 94°C for 15 sec and 60°C for 1 min. The soybean RNA helicase [34] was used as an endogenous control to normalize the RT-PCR values. Gene expression was quantified by using the 2^−ΔCT^ or 2^−ΔΔCT^ method. The data were subjected to statistical analysis, and the means were compared using confidence intervals generated by the t test at P≤0.05.

## Results

### Leaf water potential in BiP-overexpressing lines under a slow soil-drying regime

**Figure 1:**
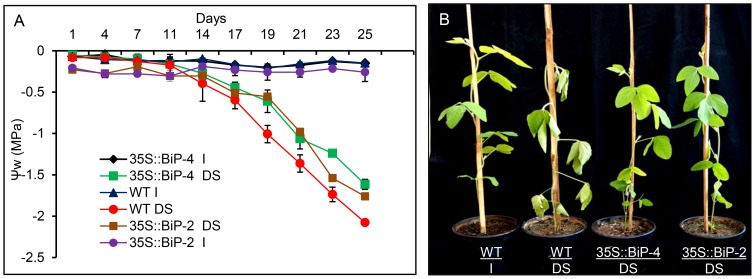
Drought-induced leaf dehydration in soybean transgenic and WT lines. (A) Drought stress was induced by reducing irrigation to 40% of the daily normal water supply. Leaf water potential was measured by using a Scholander pump. The leaf ψ_w_ of stressed wild-type plants declined to a maximum stress of −2.0 MPa, whereas the leaf ψ_w_ of transgenic plants did not decrease below −1.5 MPa. The bars represent confidence intervals (p≤0.05, n = 4). (B) Leaves were photographed on day 19 of the experiment, when 35S::BiP-4 and 35S::BiP-2 leaves displayed ψw = −0.6 MPa, whereas WT leaves presented a ψw = −1.0 MPa.

The soybean transgenic lines 35::BiP-2 and 35S::BiP-4 overexpress *soyBiPD* (GeneBank accession AF031241) [41] under the control of the CaMV 35S promoter [33] and they were subjected to a slow soil-drying treatment in which a water deficit was induced by reducing irrigation to a 40% level for 25 d. The leaf ψ_w_ of WT and BiP-overexpressing lines was progressively monitored at the third emergent trifoliate at dawn using a Scholander pump [42]. The differences between transformed and non-transformed plants were registered after 19 d under the restricted water regime, in which the irrigation was reduced to 40% of the daily normal water supply (Figure 1B). The BiP-overexpressing lines had normal turgid leaves (ψ_w_ = −0.6 MPa), whereas the WT lines displayed completely wilted leaves (ψ_w_ = −1.0 MPa) when photographed at 10 am. After 25 d under progressive dehydration, the leaf ψ_w_ of stressed WT plants declined to a maximum stress of −2.0 MPa, whereas the leaf ψ_w_ of transgenic plants did not decrease below −1.6 MPa (Figure 1A). The leaf water status indicated that the BiP-overexpressing lines were protected against dehydration; the decline in the leaf ψ_w_ was delayed in these lines under a slow water deficit regime.

### Dehydration-induced transcriptomes from WT and 35S::BiP-4 leaves

The previous experiment clearly demonstrated that BiP overexpression delays leaf dehydration under a slow soil drying treatment. However, whether BiP maintains cellular homeostasis and prevents the establishment of endogenous stress under water deficit conditions remained to be determined. To address this issue, we performed microarray analyses to compare the transcriptome changes in drought-stressed 35S::BiP-4 leaves and drought-stressed WT leaves vs well-watered WT leaves at the V3/V4 transition stage to delineate the BiP-induced changes under drought conditions. We also analyzed the drought-induced changes in 35S::BiP-4 leaves by comparing the transcriptome changes of drought-stressed 35S::BiP-4 leaves vs well-watered 35S::BiP-4 leaves. Affymetrix Soybean Array GeneChips (61K) were hybridized with cRNAs from 35S::BiP-4 leaves at ψ_w_ = −1.0 MPa and ψ_w_ = −1.6 MPa, which corresponded to 21 and 25 days post-treatment [Figure 1A; 35S::BiP-4 (ψ_w_ = −1.0) 21d and 35S::BiP-4 (ψ_w_ = −1.6) 25d], respectively, and from WT leaves at ψ_w_ = −1.0, ψ_w_ = −1.7 and ψ_w_ = −2.0 MPa, which corresponded to 19, 23 and 25 days post-treatment, respectively, in addition to cRNAs from well-watered 35S::BiP-4 leaves and well-watered WT leaves. These last two treatments served as the reference controls and allowed us to examine the BiP-induced differential gene expression compared to wild-type leaves under normal, well-watered conditions. The results were analyzed with R/Bioconductor (Version 1.6/Version 2.6.0) [43] and the data were normalized by using the Robust Multiarray Analysis (RMA) method [44]. Each treatment was compared with the control, irrigated WT leaves [35S::BiP-4(ψ_w_ = −1.0)21d/WTirrigated and 35S::BiP-4(ψ_w_ = −1.6)21d/WTirrigated; WT(ψ_w_ = −1.0)19d/WTirrigated, WT(ψ_w_ = −1.7)23d/WTirrigated and WT(ψ_w_ = −2.0) 25d/WTirrigated]. The transcriptome of stressed 35S::BiP-4 leaves was also compared with irrigated, well-watered 35S::BiP-4 leaves [35S::BiP-4(ψ_w_ = −1.0)21d/35S::BiP-4irrigated and 35S::BiP-4(ψ_w_ = −1.6)21d/35S::BiP-4irrigated]. The quality of the array was assessed with pairwise comparisons using MA plots (Figure S1).

**Figure 2:**
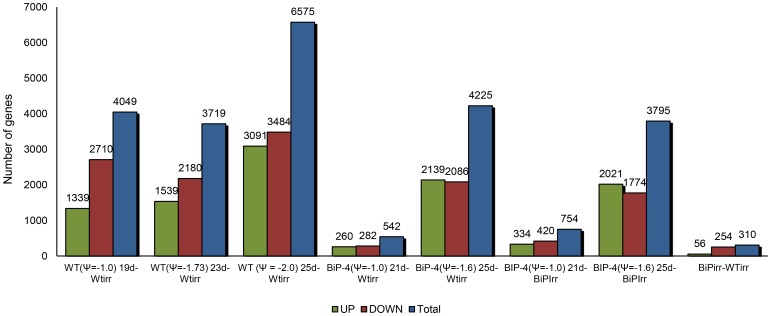
Global variation in gene expression induced by drought in the BiP-overexpressing line 35S::BiP-4. The plots indicate the number of differentially expressed genes in BiP-overexpressing leaves at ψ_w_ = −1.0 and ψ_w_ = −1.6, which corresponded to 21 and 25 days under a water deficit [35S::BiP-4 (ψ_w_ = −1.0) 21d and 35S::BiP-4 (ψ_w_ = −1.6) 21d], respectively and WT leaves at ψ_w_ = −1.0, ψ_w_ = −1.7 and ψ_w_ = −2.0, which corresponded to 19, 23 and 25 days under water stress, respectively, compared to well-watered WT leaves and well-watered BiP-overexpressing leaves.

By using a corrected p-value<0.05 and log2-fold-change >1.5 for up-regulated genes and log2-fold-change<−1,5 criteria for down-regulated genes, we observed that a decline in the WT leaf ψ_w_ to −1.0 MPa was enough to induce robust gene expression reprogramming in WT leaves, but not in 35S::BiP-4 leaves (Figure 2). In both WT and 35S::BiP-4 lines, the number of differentially expressed genes increased with the severity of the drought stress. When compared under the same water potential (−1.0 MPa), drought-stressed WT leaves displayed 4049 differentially expressed genes, whereas in the BiP-overexpressing line only 754 genes were differentially expressed. This scenario did not persist with stress progression because a comparison between the drought-induced transcriptomes of WT leaves at −1.7 MPa and 35S::BiP-4 leaves at −1.6 MPa revealed that the number of differentially expressed genes in 35S::BiP-4 (4225) was slightly superior to that of WT leaves (3719), when compared to well-watered WT (WTirr). When we compare the transcriptome changes of 35S::BiP-4(ψ_w_ = −1.6)21d vs well-watered 35S::BiP-4 leaves, the number of differentially expressed genes in 35S::BiP-4 decreased to 3795 and was similar to that of stressed WT(ψ_w_ = −1.7)23d leaves. These results collectively indicated that BiP overexpression not only delayed (19 d×21 d) the drought-mediated decline in leaf ψ_w_, but it also attenuated the establishment of endogenous stress induced by a −1.0 MPa leaf ψ_w_. This level of leaf dehydration induced a modest change in the gene expression of 35S::BiP-4 leaves relative to the robust changes displayed by WT leaves at ψ_w_ = −1.0 MPa. These results are consistent with previous observations indicating that BiP-overexpressing lines display attenuated stress symptoms when they are exposed to a reduction in the daily water supply or to treatments with osmotic stress inducers [31]–[34], [39]. However, under a more severe stress conditions (ψ_w_ = −1.6 to −1.7), BiP overexpression may not alleviate the effect of drought in the transgenic lines.

**Figure 3:**
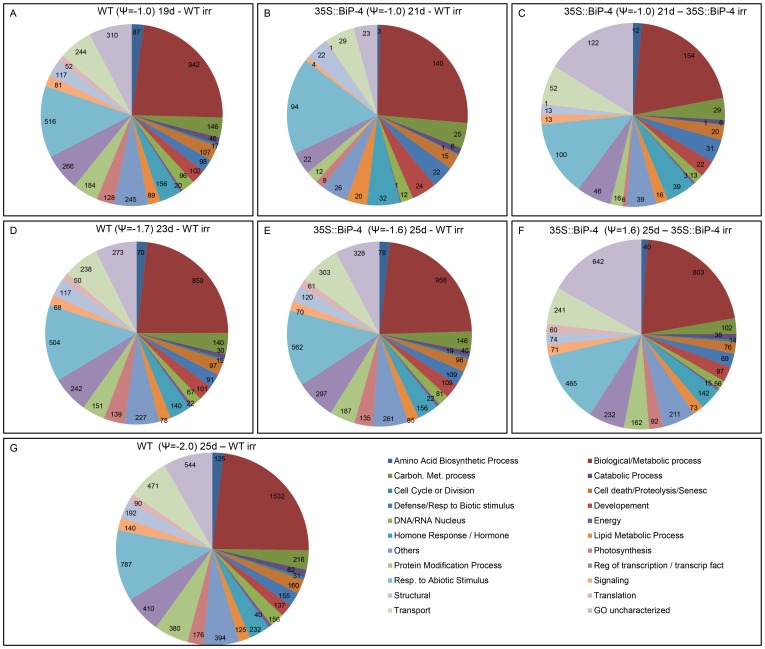
Functional categorization of differentially expressed genes based on biological processes. The pie chart illustrates the distribution of differentially expressed genes across functional categories as defined by the Gene Ontology Biological process. The water potential and days after treatment are indicated in the figure. The numbers represent the frequency of differentially expressed genes in each category compared to the well-watered WT control.

Water deficits induce a complex network of adaptive responses in plants, which begins with the stress perception that activates molecular events leading to physiological, metabolic and developmental changes to improve defenses against stress conditions. To facilitate the interpretation of results, differentially expressed genes were functionally categorized via Gene Ontology (http://amigo.geneontology.org/); some of the categories were condensed to allow for a graphical representation of the data (Figure 3).

In the amino acid metabolism category, the down-regulated changes predominated over the up-regulated changes in all treatments (Figures 3A, 3D, 3G, and Tables S2 and S3). WT(ψ_w_ = −1.0)19d, WT(ψ_w_ −1.7)23d and WT(ψ_w_ −2.0)25d leaves displayed a massive number of differentially expressed genes (ranging from 70 to 125 genes) in comparison to well-watered leaves. In addition to proline, which functions as an osmolyte to alleviate dehydration-mediated cellular damages [47], under moderate water stress, other amino acids, such as valine, arginine and leucine, accumulate at high levels in response to ABA [48]. Our results indicate that drought-induced leaf dehydration to ψ_w_ = −1.0 MPa is enough to efficiently alter the amino acid metabolism of WT leaves. In contrast, only 3 to 12 genes from this category were differentially expressed in dehydrated 35S::BiP-4 leaves at ψ_w_ = −1.0 MPa (Figures 3B, 3C), indicating that the BiP-overexpressing line maintained normal levels of basal leaf metabolism under moderate stress conditions. However, the number of differentially expressed amino acid metabolism genes under more severe stress conditions (ψ_w_ −1.6 MPa) in dehydrated 35S::BiP-4 leaves were similar to the number of ψ_w_ = −1.7 MPa-induced changes in WT leaves. Likewise, in all other functional categories, the number of ψ_w_ = −1.0 MPa differentially regulated genes was very low in 35S::BiP-4 leaves in contrast to WT leaves, in which drought-induced changes by a leaf ψ_w_ = −1.0 MPa reached levels similar to those induced by ψ_w_ = −1.7 MPa in WT leaves and ψ_w_ = −1.6 MPa in 35S::BiP-4 leaves (Figure 3).Therefore, under progressive dehydration, a moderate stress (ψ_w_ = −1.0 MPa) induces a robust global variation in gene expression in WT leaves and this variation persisted at similar level through ψ_w_ = −1.7 MPa (intermediate level of stress). These stress levels may correspond to an acclimation period in which rapid adaptive responses emerge to cope with dehydration. However, under a severe stress condition of ψ_w_ = −2.0 MPa, the WT leaves displayed a further increase in differentially expressed genes in all functional categories (Figure 3G, Table S2). These results revealed that progressive leaf dehydration induces a robust increment in the number of differentially expressed genes in soybean leaves at a moderate stress level of ψ_w_ = −1.0 MPa. This expression plateaus in response to intermediate stress conditions (ψ_w_ = −1.7 MPa), followed by a further increment in the number under severe stress conditions (ψ_w_ = −2.0 MPa). In contrast, a two-slope change in global gene expression was not observed in the transgenic line, and a robust change in the number of differentially expressed genes was only evident with a decline in the leaf ψ_w_ to −1.6 MPa. Collectively, these results further substantiate the argument that BiP protects plant cells against dehydration-induced stress.

In both WT and 35S::BiP-4 leaves, the number of down-regulated photosynthesis-related genes increased as the stress persisted, but, nevertheless, these down-regulated changes were lower in stressed transgenic lines, mainly in 35S::BiP-4(ψ_w_ = −1.0)21d (Figure 3, Tables S2 and S3). These findings are consistent with recently published results revealing that drought induces a down-regulation of photosynthesis-related genes in soybean leaves [49], which is parallel to the inhibition of photosynthetic rates displayed by drought-stressed soybean plants [33]. The inhibition of photosynthesis under drought conditions leads to stunted growth and this response has been proposed to serve as an adaptive plant survival mechanism in stress conditions [49]. Our results may also invoke an alternative mechanism for BiP protection against dehydration because the down-regulation of photosynthesis-related genes under drought conditions was attenuated in 35S::BiP-4 leaves. Accordingly, the drought-induced decrease in the photosynthesis rate has been shown to be significantly lower in BiP-overexpressing lines than in WT leaves [33], [39].

Among the differentially expressed genes, abiotic stimuli-responsive genes predominated in all treatments. In this functional category, the number of differentially expressed genes increased as the stress persisted and except for WT(ψ_w_ = −1.0)19d in which the down-regulated genes predominated, in all other treatments the number of induced genes was superior (Tables S2 and S3). This functional category included functional drought-responsive genes, such as HSPs, LEA, peroxidases, protease inhibitors and drought-responsive regulatory genes, which were predominantly listed in the transcription factor category (Tables S4, S5, S6, S7, S8, S9, S10, S11, S12, S13).

A close inspection of the abiotic-responsive genes revealed that those genes are regulated by a variety of primary stresses, such as drought, heat, cadmium, cold, salt, which generates an oxidative stress signal as a common secondary stress. The BiP- induced transcriptome in leaves of well-watered leaves revealed that the most significant enriched GO term among the 254 BiP-mediated down-regulated genes (Figure 2) belongs to the abiotic stimulus-responsive category with a large proportion of dehydration-induced genes and components of the antioxidant system (Table S14) [50]. This finding may explain the large difference observed in the number of differentially expressed abiotic-responsive genes mediated by BiP overexpression in stressed leaves [35S::BiP-4(ψ_w_ = −1.6)21d/WTirrigated; 593 differentially expressed genes, Table S2] and induced by drought in stressed 35S::BiP-4 leaves [35S::BiP-4(ψ_w_ = −1.6)21d/35S::BiP-4irrigated; 465 differentially expressed genes, Table S3]. Under normal conditions, BiP overexpression also down-regulated protein degradation and cell death-associated genes (Table S14), which may be consistent with the negative effects of BiP on stress-induced senescence or cell death programs [31]–[33]. Our genome-wide expression analysis also suggested that BiP inhibited drought-induced senescence and cell death-associated genes, which were mostly down-regulated in stressed 35S::BiP leaves.

**Figure 4:**
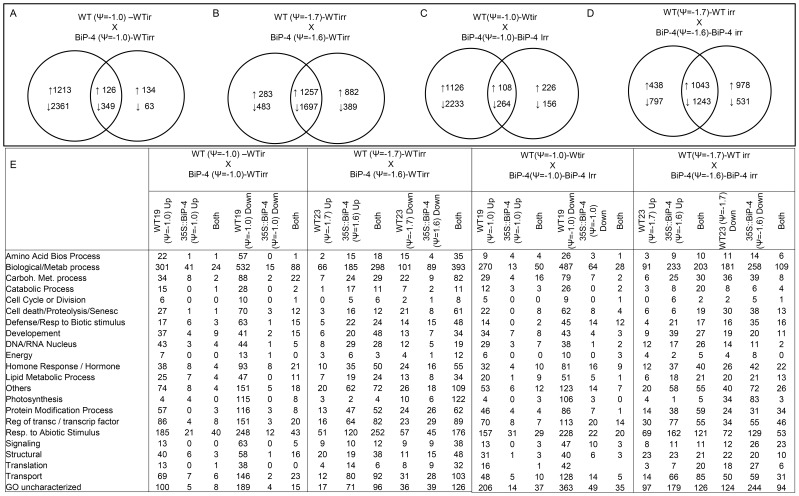
A Venn diagram representing the distribution of shared and specific responses induced by progressive drought in WT and 35S::BiP-4 plants under a similar leaf Ψw. A. The number of shared and specific differentially expressed genes by comparing (A) WT (Ψw = −1.0)-WTirr and 35S::BiP-4 (Ψw = −1.0)- WTirr, (B) WT (Ψw = −1.0)-WTirr and 35S::BiP-4 (Ψw = −1.0)- 35S::BiP-4irr, (C) WT (Ψw = −1.7)-WTirr and 35S::BiP-4 (Ψw = −1.6)-WTirr and (D) WT (Ψw = −1.7)-WTirr and 35S::BiP-4 (Ψw = −1.6)-35S::BiP-4irr. The arrows indicate up-regulated (↑) and down-regulated (↓) genes. (E) The number of up- and down-regulated genes in each functional category based on Biological Processes through *Gene Ontology*.

The drought-induced activation of transcriptional factors occurs with rapid kinetics response to dehydration stress. In fact, a robust activation of bZIP, MYB, WRKY and NAC genes was evident in the WT at a leaf water status as low as ψ_w_ = −1.0 MPa (Table S3). A large repertoire of drought-induced NAC genes was already activated under these conditions and the NAC transcripts accumulated to higher levels as the stress progressed to a lower water potential (Table S15). In contrast, the induction of transcriptional factors in 35S::BiP-4 leaves was only evident at a stress level of ψ_w_ = −1.6 MPa (Figure 4C, Tables S2 and S3), indicating that BiP may delay the perception of dehydration-induced endogenous stress.

Abiotic stress causes the inhibition of plant growth and development, a reduction in photosynthesis and respiration and disturbances in nucleic acid metabolism [51], [52]. One of the most rapid responses to drought acclimation is the decrease in leaf growth. In Arabidopsis, the inhibition of leaf growth is compensated by a slight induction of expansin genes, which causes an increase in cell expansion to reduce the transpiration area and thereby maintain the leaf turgor [53]. In the 35S::BiP-4 line, a decline in the leaf water potential to −1.0 MPa induced expansin genes to a higher extent, which may contribute to the maintenance of leaf turgidity for a longer period under dehydration, a typical phenotype of BiP-overexpressing lines [Table S10 (B)].

### Identification of common and specific up- and down-regulated changes between WT and 35S::BiP-4 lines under a gradual water deficit regime

The differentially expressed genes identified by comparing the WT(ψ_w_ = −1.0)xWTirr, 35S::BiP-4(ψ_w_ = −1.0)xWTirr, 35S::BiP-4(ψ_w_ = −1.0)×35S::BiP-4irr, WT(ψ_w_ = −1.7)xWTirr,35S::BiP-4(ψ_w_ = −1.6)xWTirr, and 35S::BiP-4(ψ_w_ = −1.6)×35S::BiP-4irr were subjected to a Venn diagram analysis to detect specific and shared up- and down-regulated changes between two comparisons. Because BiP overexpression seems to relieve dehydration-induced cellular stress, the drought-induced transcriptome between the WT and 35S::BiP-4 lines were compared by observing similar levels of leaf water potential, as in WT(ψ_w_ = −1.0)-WTirr×35S::BiP-4(ψ_w_ = −1.0)-WTirr, WT(ψ_w_ = −1.0)-WTirr×35S::BiP-4(ψ_w_ = −1.0)-35S::BiP-4irr, WT(ψ_w_ = −1.7)-WTirr×35S::BiP-4(ψ_w_ = −1.6)-WTirr and WT(ψ_w_ = −1.7)-WTirr×35S::BiP-4(ψ_w_ = −1.6)-35S::BiP-4irr (Figure 4). The WT(ψ_w_ = −1.0)×35S::BiP-4(ψ_w_ = −1.0) comparison resulted in the overlap of 126 up-regulated and 349 down-regulated genes in both treatments. The WT(ψ_w_ = −1.0)-specific changes consisted of 1213 up-regulated and 2361 down-regulated genes, whereas a much lower number of differentially regulated genes was specifically identified in 35S::BiP-4(ψ_w_ = −1.0) leaves (Figures 4A and 4C). Those genes were grouped based on functional categories and presented in Figure 4E and Tables S16, S17, S18, S19, S20, S21, S22, S23.

In the amino acid metabolism group, we specifically identified 22 up-regulated genes in WT (ψ_w_ = −1.0) leaves that were involved in both catabolic and anabolic processes and 57 down-regulated genes, among which 41 genes were related to the biosynthesis of amino acids (Figure 4E; Tables S17 and S19). In these stressed plants, the number of carbohydrate metabolism-related genes were also considerably altered by the water status of a leaf ψ_w_ = −1.0 MPa with 34 up-regulated and 88 down-regulated genes. In the up-regulated changes, the major proportion (23 genes) was attributed to catabolic process-related genes, i.e., oligosaccharide hydrolysis (Tables S17 and S19). In the cell death and proteolysis functional category, the down-regulated genes (82) largely predominated over the up-regulated changes (28 genes; Tables S17 and S19). Nevertheless, programmed cell death (PCD)-related genes, such as caspase-like encoding genes *CASP-like protein 3* (Glyma13g01100); *CASP-like protein 8* (Glyma07g38110), C*ASP-like protein 10-like* (Glyma11g33030) and cysteine proteinase (Glyma10g35100), were specifically induced in the WT (ψ_w_ = −1.0) leaves. The down-regulation of proteolysis-, anabolic process- and cell death-related genes along with the induction of PCD-related genes may serve as an adaptive mechanism in soybean plants to survive under moderate stress at ψ_w_ = −1.0 MPa. In contrast, under the same leaf ψ_w_ (−1.0 MPa) conditions, the 35S::BiP-4 leaves displayed only slight alterations in the number of amino acid metabolism-related genes and carbohydrate metabolism-related genes, with a predominance of sucrose and starch biosynthetic genes and proteolysis- and cell death-related genes (8 drought-induced and 12 drought-repressed genes, Table S3, Figure 4E, Tables S16 and S18). In this latter category, we identified vacuolar processing enzyme 2 (Glyma14g10620), a key player in plant-specific PCD [54], which were specifically repressed in 35S::BiP-4 leaves. These results are consistent with previous reports revealing that BiP modulates stress-induced PCD in plants [31], [33].

In all the other categories, the WT(ψ_w_ = −1.0)-specific changes were also much higher than those displayed by 35S::BiP-4(ψ_w_ = −1.0), which may reflect the BiP capacity to maintain cellular homeostasis and prevent the build-up of endogenous stress under moderate leaf dehydration. The WT(ψ_w_ = −1.0)-specific changes included (i) regulatory drought-responsive genes (86 up-regulated and 151 down-regulated), such as the bZIP, WRKY, MYB, bHLH and NAC transcriptional factors; (ii) functional drought-responsive genes (80), such as LEA, dehydrins, HSPs and peroxidases; and (iii) hormone-related genes (38 induced and 93 repressed genes; Tables S17 and S19). This scenario illustrates the major drought-induced changes previously reported in drought-stressed soybean leaves [49], confirming that a decline in the leaf ψ_w_ to −1.0 MPa is enough to induce typical drought-responsive changes in WT leaves but not in 35S::BiP-4 leaves. However, by raising the stress level to ψ_w_ = −1.6 in 35S::BiP-4 leaves, the number of differentially expressed genes increased to an extent similar to that in the WT (ψ_w_ = −1.7) leaves with a concomitant increase in the number of overlapping differentially expressed genes and an accentuated decrease in the WT(ψ_w_ = 1.7)-specific changes (Figure 4). In fact, 1257 up- and 1697 down-regulated genes represented the shared response in the WT(ψ_w_ = −1.7)-WTirr×35S::BiP-4(ψ_w_ = −1.6)-WTirr comparison (Figure 4B). The number of overlapping genes decreases to 1043 up- and 1243 down-regulated genes with a concomitant increase in the specific changes when we examined the WT(ψ_w_ = −1.7)-WTirr×35S::BiP-4(ψ_w_ = −1.6)-35S::BiPirr comparison (Figure 4C). This number variation indicates that drought induces specific changes in 35S::BiP-4 (ψ_w_ = −1.6) stressed leaves, which may account for the slight better adaptation displayed by the transgenic lines under a higher dehydration status These genes were grouped into functional categories (Tables S19, S20, S21, S22, S23) and the size of each category was represented in Figure 4E. Taken together, these results indicate that BiP overexpression protects plant cells against the deleterious effects of leaf dehydration by preventing cellular homeostasis disruption at a low leaf ψ_w_ rather than by inducing a specific sub-set of drought resistance genes.

### The differential expression of genes involved in abiotic stress in BiP-overexpressing leaves

To analyze the global variation of drought-induced genes in specific categories, we used Gene Ontology along with Glyma (www.phytozome.com) to select genes and generate heatmaps with absolute expression values resulting from the microarray hybridization. Because BiP is an ER-resident molecular chaperone that has been shown to regulate UPR [30] and cell death response [31], we also examined genes involved in UPR, ERAD and cell death in addition to drought-responsive genes.

**Figure 5:**
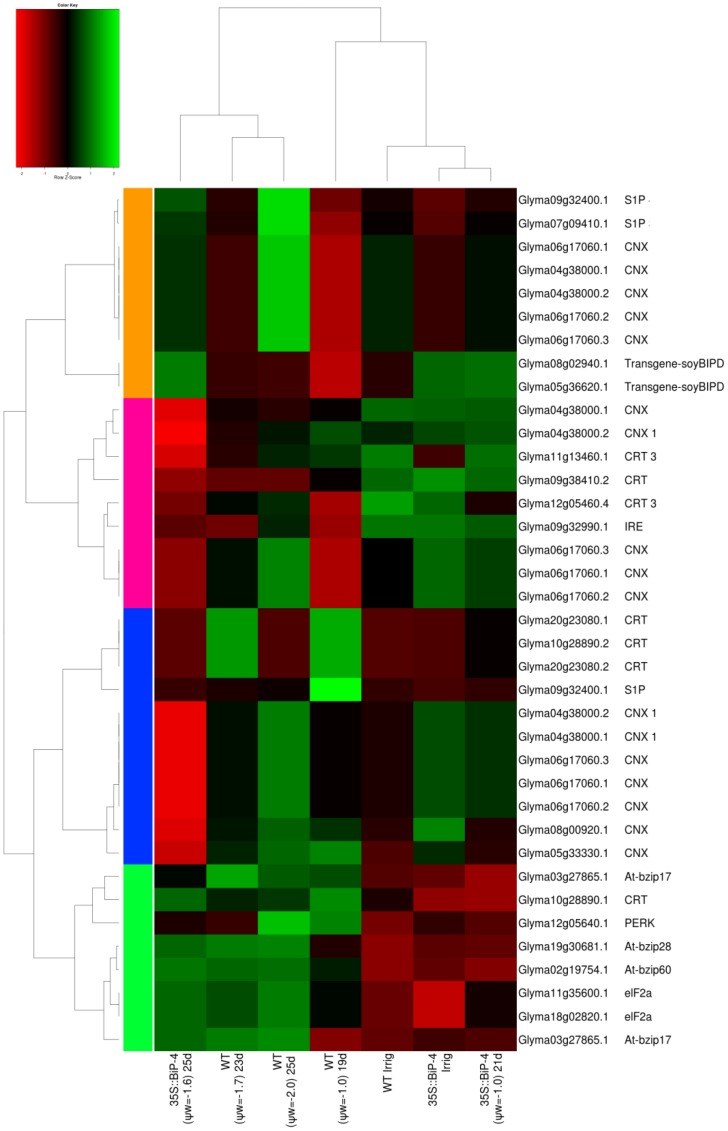
Heatmap of UPR genes. The data presented in the figure comprise the microarray measurements from well-watered and stressed leaves (with different leaf water potential) from WT and 35S::BiP-4 plants under progressive drought.

**Figure 6:**
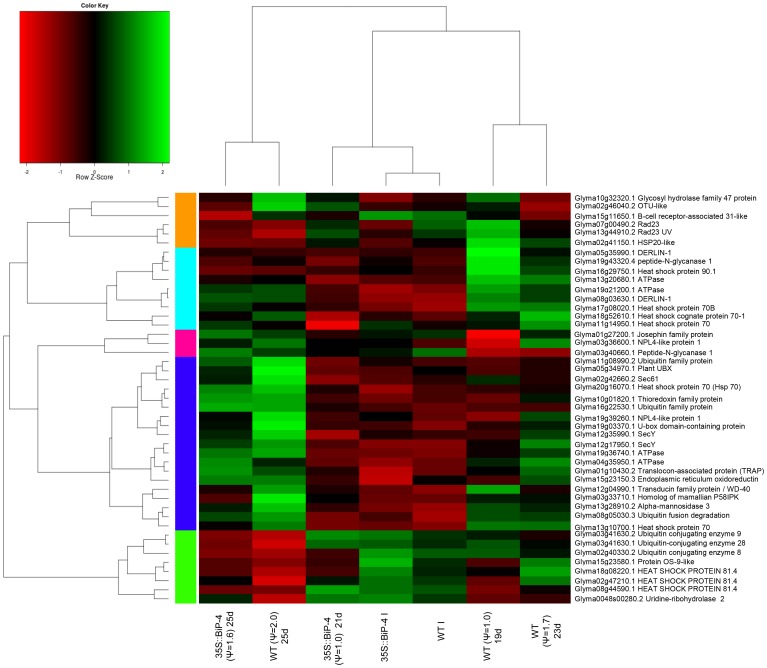
Heatmap of ERAD genes. The data presented in the figure comprise a microarray measurement of well-watered and stressed leaves (with different leaf water potential) from WT and 35S::BiP-4 plants under progressive drought.

A close inspection of the UPR-regulated genes revealed that the transcriptome displayed by 35S::BiP-4(ψ_w_ = −1.0) leaves was most closely related to the WTirr and 35S::BiP-4irr transcriptomes as they clustered together for the global comparison and they differed greatly from the WT(−2.0) and 35S::BiP-4(ψ_w_ = −1.6)-induced transcriptomes. A higher accumulation of BiP transgene transcripts was evident in transgenic lines (Figure 5). The heatmap indicates a tendency towards negative regulation in the UPR genes CNX and CRT in 35S::BiP-4 under ψ_w_ = −1.6. We identified a tendency for the positive regulation of bZiP28, S1P and CNX in WT leaves as the stress increases to −2.0 MPa, indicating that UPR is activated by dehydration. As downstream components of UPR, the genes involved in ERAD displayed an expression profile similar to that of the UPR genes (Figure 6). In fact, the transcriptome of 35S::BiP-4(ψ_w_ = −1.0) was grouped with WTirr and 35S::BiP-4irr transcriptomes and they were separated from the cluster of WT(ψ_w_ = −2.0) and 35S::BiP-4(ψ_w_ = −1.6)-induced transcriptomes.

**Figure 7:**
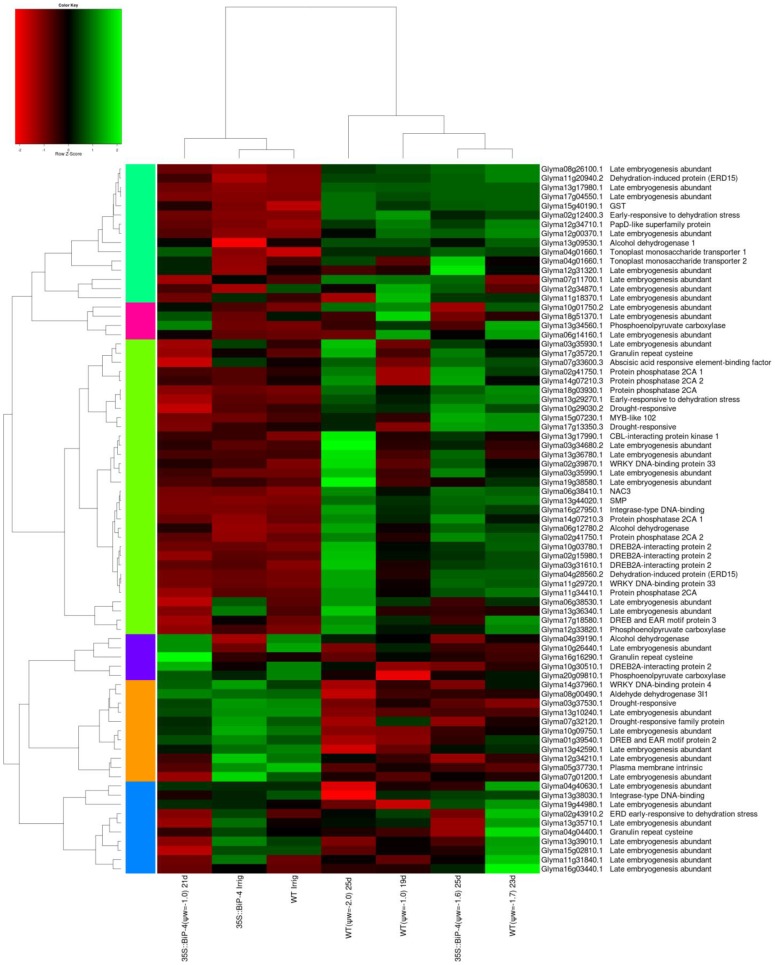
Heatmap of the functional and regulatory drought-responsive genes involved in protection. The data presented in the figure comprises microarray measurements of well-watered and stressed leaves (with different water status) from WT and 35S::BiP-4 plants under progressive drought.

**Figure 8:**
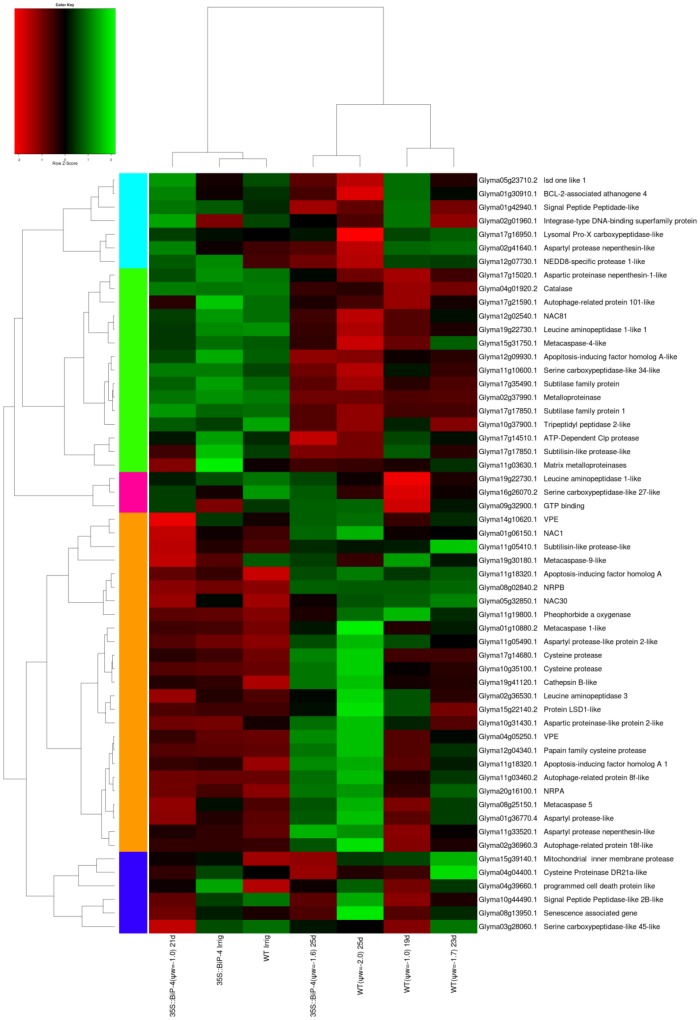
Heatmap of cell death-associated genes. The data presented in the figure comprises microarray measurements of well-watered and stressed leaves (with a different water status) from WT and 35S::BiP-4 plants under progressive drought.

The drought-responsive genes (Figure 7) and cell death-associated genes (Figure 8) also displayed a similar expression profile as that of UPR-related genes in response to a gradual drought regime. In these categories, the transcriptomes of WTirr, 35S::BiP-4irr and 35S::BiP-4(ψ_w_ = −1.0) were closely related and formed a separate clade from WT(ψ_w_ = −1.0), 35S::BiP-4(ψ_w_ = −1.6), WT(ψ_w_ = −1.7) and WT(ψ_w_ = −2.0)-induced transcriptomes. The relatedness of WTirr and 35S::BiP-4(ψ_w_ = −1.0) drought-responsive transcriptomes further indicates that BiP maintains cellular homeostasis under moderate leaf dehydration stress. In WT leaves, the up-regulation of regulatory genes (DREB2A, NACs, ERD15, ERD4 and phosphatases) and functional drought responsive genes (LEA, dehydration-induced proteins, aldehyde dehydrogenase and dehydrin) were increased under progressive stress (Figure 7). In the 35S::BiP-4 leaves, the induction of these drought-responsive genes were much lower and only apparent when the stress level reached ψ_w_ = −1.6 MPa. Likewise, the majority of differentially expressed cell death-associated genes were more strongly induced under progressive stress in WT leaves than in 35S::BiP-4 (Figure 8), which is consistent with the BiP capacity of modulating stress-induced cell death in plants [31], [33]. Collectively, these results indicate that the ectopic expression of BiP prevents the establishment of endogenous dehydration-mediated stress in plant cells, because the leaf transcriptome of 35S::BiP-4 at ψ_w_ = −1.0 MPa was similar to that of the control leaves under normal irrigation and contrasted with the dramatic variation displayed by the drought-induced transcriptome of WT leaves at ψ_w_ = −1.0 MPa. In the 35S::BiP-4 line, the induction of typical drought-responsive genes that presumably protect plant cells against dehydration (Figure 7) occurred only at a lower leaf water potential (ψ_w_ = −1.6) and to a lower extent than in WT leaves under similar leaf dehydration conditions (ψ_w_ = −1.7). However, it is not surprising that the drought-induced expression profiles of UPR, ERAD and cell death-associated genes were similar to those of the drought-responsive genes because BiP has been shown to inhibit the activation of UPR/ERAD and to attenuate stress-induced cell death in plants [28]–[33].

**Figure 9:**
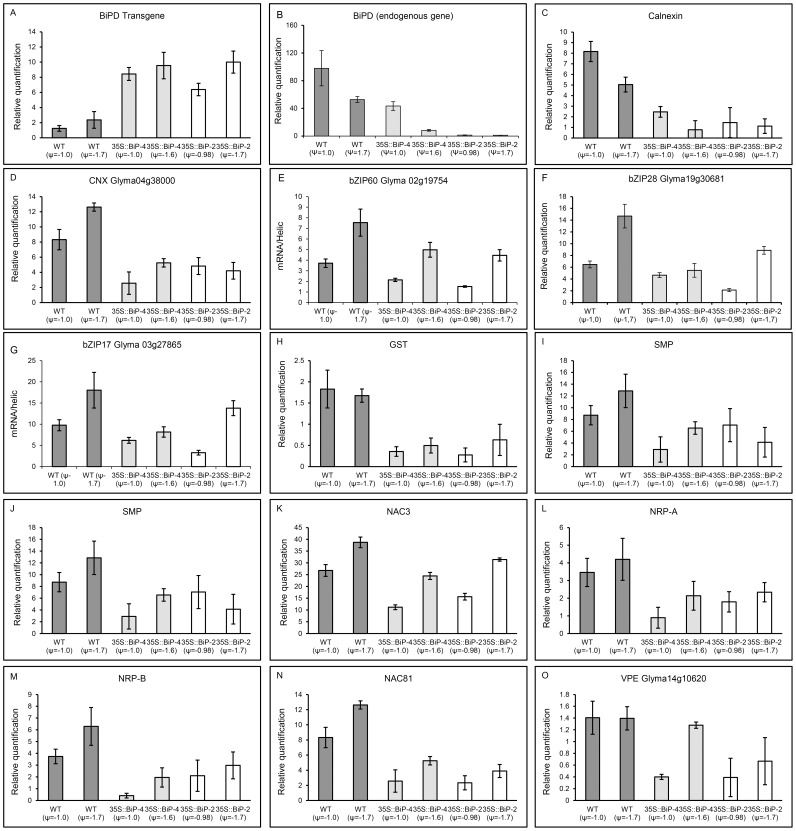
Transcript accumulation for UPR marker genes, cell death-associated genes and drought-responsive genes in WT and BiP-overexpressing lines under progressive drought. The total RNA was isolated from WT, 35S::BiP-4 and 35S::BiP-2 water-stressed leaves and the expression of the transgene (A), UPR marker genes B–G), drought-responsive genes (H–K) and cell death-associated genes (L–O) was monitored by qRT-PCR. The gene expression was calculated by using the 2^−ΔCT^ method and RNA helicase was used as an endogenous control. The bars indicate the confidence interval based on the t test (p<0.05, n = 3).

The microarray and heatmap results for the differential expression of selected stress-responsive genes were confirmed by quantitative RT-PCR. For this assay, we also used a second independently transformed BiP-overexpressing line, called 35S::BiP-2, which has also been shown to be tolerant of water deficits [33]. A gene expression analysis by qRT-PCR first confirmed the high levels of *soy*BiPD transgene accumulation in transgenic lines 35S::BiP-4 and 35S::BiP-2 (Figure 5, Figure 9A). Progressive drought induced the UPR marker genes, CNX (Glyma04g38000 and Glyma05g33330), endogenous BiP and bZIP60 (Figures 9B, 9C, 9D and 9E). We also examined the UPR transducers bZIP28 and bZIP17 as they were induced by drought in the UPR heatmap (Figure 5). The quantititative RT-PCR results confirmed the drought induction of the UPR marker genes (Figures 9B–9G) Nevertheless, the ectopic expression of BiP inhibited the drought induction of UPR marker genes, because the UPR transcript level was lower in the transgenic lines than in the WT under the same leaf ψ_w_ status (Figure 5, Figures 9B–9G),). These results confirmed that gradual dehydration stress induces UPR in the WT line, and BiP overexpression negatively impacts the drought-induced activation of UPR gene expression. The expression of representative drought-responsive gene samples (Figure 7; Tables S2 and S3), including the functional genes glutathionae-S-transferase (GST; Glyma15g40190) [34], seed maturation protein (SMP; Glyma13g44020) [34], and the regulatory genes NAC2, (Glyma06g11970) [53], also designated GmNAC35, and NAC3 (Glyma06g38410) [28], also designated GmNAC043, were also examined by qRT-PCR. Consistent with the microarray results (Tables S2 and S3, Figure 7), progressive drought induced the expression of these drought-responsive genes (Figures 9H–9K). However, the induction of drought-responsive genes in 35S::BiP-4 and 35S::BiP-2 lines was lower than in WT leaves under a similar leaf water potential status. These results are congruent with our data on the global variation of gene expression and confirmed that BiP overexpression inhibits the induction of functional and regulatory drought-responsive genes, suggesting that BiP may alleviate endogenous dehydration stress.

Because BiP has been shown to inhibit the osmotic and ER stress-induced cell death signaling pathway [31] (Figure 8), we compared the expression of cell death signaling components NRP-A and NRP-B [28], NAC81 [55] and VPE [37] under a progressive water deficit. In the WT, the decline of the leaf ψ_w_ to −1.0 MPa or −1.7 MPa induced the expression of NRP-A, NRP-B, GmNAC81 and the VPE homolog Glyma14g10620, (Figures 9L–9O). Consistent with the negative role for BiP in modulating the stress-induced cell death pathway, the expression of cell death signaling components in both 35S::BiP-2 and 35S::BiP-4 leaves were repressed in response to a decline in the leaf ψ_w_ to −1.0 MPa and were modestly induced by a further decrease in the leaf ψ_w_ to −1.6 MPa (Figures 6 and 8). A down-regulation of VPE transcripts in 35S::BiP-4 (−1.0) and 35S::BiP-4 (−0.99) plants was also detected in the microarray results (Table S18). In addition to confirming the microarray hybridizations, the qRT-PCR results revealed that BiP overexpression similarly affected the UPR marker genes, cell death-associated genes and drought-responsive genes from independently transformed lines 35S::BiP-4 and 35S::BiP-2.

## Discussion

### Enhanced BiP accumulation maintains cellular homeostasis under water deficit conditions

BiP overexpression has been demonstrated to confer drought tolerance in tobacco [39] and, more recently, in soybeans [33]. In addition to a more efficient photosynthetic rate, the maintenance of leaf turgidity is prolonged in BiP-overexpressing plants when compared with WT lines under the same water deficit regime. To determine whether BiP-mediated protection against leaf dehydration is associated with a delay in the decline of leaf water potential under progressive drought or the amelioration of endogenous stress, the BiP-overexpressing and WT leaf transcriptomes induced by a slow soil drying regime were evaluated under similar levels of leaf ψ_w_ (similar RWC). As previously observed, exposing soybeans to a gradual dehydration regime caused a slower decline of the leaf ψ_w_ of the 35S::BiP-4 and 35S::BiP-2 lines than in WT leaves, which maintained the leaf turgor for a longer period (Figures 1A and 1B). An analysis of global gene expression variations by gradual leaf dehydration clearly demonstrated that the leaf water status of ψ_w_ = −1.0 MPa is low enough to promote extensive gene expression reprogramming in soybean leaves. These results indicate that leaf ψ_w_ = −1.0 induces endogenous cellular stress that is efficiently sensed by leaf cells to activate typical responses to dehydration stress, such as the up-regulation of typical drought-responsive regulatory and functional genes (*DREB*, *NAC*, *AREB*, *LEA*, *GST*, peroxidases, etc) as well as the down-regulation of photosynthesis-related genes. This scenario of high variations in the number of differentially expressed genes is consistent with the drought-induced transcriptome identified by Le et al. [49] in soybean leaves exposed to drought. In contrast, the 35S::BiP-4 leaves at ψ_w_ = −1.0 MPa displayed a considerably lower number of differentially expressed genes. These results indicate that BiP overexpression prevents variations in gene expression as induced by a leaf water status of ψ_w_ = −1.0 MPa, most likely because of its ability to maintain a degree of cellular homeostasis that ensures a lower level of cellular stress compared to WT leaves at the same leaf ψ_w_.

BiP-mediated ER homeostasis maintenance has been previously demonstrated in tobacco [29], [39] and mammalian cells [38]. In these studies, elevated levels of BiP attenuate the ER stress induced by misfolded protein accumulation in the lumen of the organelle. Likewise, BiP overexpression in soybeans and tobacco confers water deficit tolerance, and several lines of evidence indicate the BiP-mediated increases in water stress tolerance may be associated with its capacity to prevent the stress-mediated disruption of cellular homeostasis. First, BiP overexpression in tobacco and soybeans does not activate typical drought avoidance and/or tolerance mechanisms [33], [39]. In fact, the BiP-mediated improvement of leaf water relations has not been associated with the stimulation of stomata closure, a reduction of photosynthesis and transpiration, an increase in leaf osmolytes or root growth stimulation under dehydration conditions. Likewise, the induction of drought-responsive genes is lower in BiP-overexpressing plants than in drought-stressed WT leaves (Figures 7 and 9). It is likely that the attenuation of typical drought-induced responses in BiP-overexpressing lines reflects a lower severity of endogenous stress, indicating that BiP prevents the dehydration-mediated disruption of cellular homeostasis in plants exposed to a slow soil-drying regime. Therefore, our current results based on an analysis of global gene expression variations provides further evidence for the capacity of BiP to prevent the establishment of drought-induced osmotic stress.

In addition to displaying lower gene expression variation by drought, the transcriptome of the 35S::BiP-4 line induced by leaf dehydration at ψ_w_ = −1.0 MPa was more closely related to that displayed by well-watered WT leaves, because they clustered together in the global comparison among the abiotic stress-responsive expressed sequences of WT and 35S::BiP-4 lines with a different leaf ψ_w_ (Figure 7). In contrast, the drought-induced transcriptome of WT leaves exposed to different levels of leaf dehydration diverged considerably from that of well-watered WT leaves as they grouped separately into distant branches of the heatmap. In this case, we identified a direct relationship between the level of leaf stress (ψ_w_ = −1.0, −1.7 and −2.0) and the distance between clusters of drought-induced transcriptomes and the well-watered WT transcriptome. These absolute expression results were consistent with the differential expression results for abiotic stress-responsive genes, which demonstrated a higher number and transcript accumulation of differentially expressed genes with the persistence and increment of endogenous stress in WT plants (Table S2). In contrast, the variation in the expression of abiotic stress-responsive genes in BiP-overexpressing lines at ψ_w_ = −1.0 MPa is quite inferior to that of WT plants under the same level of endogenous stress, substantiating the argument that BiP alleviates dehydration-induced stress by maintaining cellular homeostasis.

Under progressive drought conditions, the number of differentially expressed genes shared by both treatments increased with the decline in the leaf ψ_w_ (Figure 4). This increase in common changes led to a decrease in the number of WT-specific up-regulated genes, most likely because of the stress severity. These results suggest that the activation of drought-responsive mechanisms in BiP-overexpressing plants is delayed in comparison to the rapid response of the WT when exposed to a similar leaf ψ_w_. As a consequence, a significant number of genes responded to drought differently in BiP-overexpressing lines. As an ER-resident molecular chaperone, BiP may ensure normal ER functioning for a longer period under stress, preventing the disruption of secretory pathway homeostasis.

### BiP attenuates the expression of drought-induced PCD executioners

The Venn diagram (Figure 4) revealed the remarkable predominance of differentially expressed genes in WT (ψ_w_ = −1.0) leaves in comparison to the barely detected gene variations in the 35S::BiP (ψ_w_ = −1.0) leaves. In the WT, drought conditions rapidly induced a variety of PCD-mediated genes, including caspases (*CASP-like proteins 3, 8* and *10*), which have been characterized in heat-induced PCD events in tobacco cells [56] and *cysteine proteinase*, which is involved in plant PCDs [57]. These results are consistent with recent studies demonstrating that PEG-induced osmotic stress promotes PCD in soybean seedlings [31]. In contrast, BiP overexpression attenuated alterations in cell death-associated gene expression under progressive drought (Figures 8 and 9), which is consistent with the BiP-mediated delay in drought-induced leaf senescence as previously reported [33]. A precedent in the literature has linked drought tolerance to the impairment of stressed leaves to undergo senescence [58]. The underlying mechanism for the BiP-mediated increase in water stress tolerance may be associated at least in part with its capacity to attenuate stress-induced cell death events. Our global gene expression variation results revealed that multiple stress-induced NRP-mediated cell death signaling is also induced by drought and can be modulated by BiP overexpression. In addition to detecting a down-regulation in the cell death signaling components VPE (at ψ_w_ = −1.0, Figure 9N and Table S18) and *Gm*NAC81 (at ψ_w_ = −1.6, Figure 9L, Table S22), NRPs were induced in the WT leaves at ψ_w_ = −1.0, but not in 35S::BiP-4 at ψ_w_ = −1.0 (Figures 9J and 9K). The BiP modulation of drought-induced expression for NRP-mediated cell death signaling components was further confirmed by qRT-PCR. VPE displays caspase 1-like activity and is an executioner of plant-specific PCD mediated by vacuole collapse [59], [60]. Recently, VPE has been shown to be a target of the transcriptional factors GmNAC30 and GmNAC81, which function in the osmotic and ER stress-induced NRP-mediated cell death response [31], [37]. NRP expression up-regulation mediated by the ER stress-, drought- and osmotic stress-induced GmERD15 induces GmNAC81 and GmNAC30, which in turn act in concert to activate the VPE promoter and expression, resulting in PCD activation. We propose that the BiP-mediated modulation of drought-induced NRP-mediated cell death signaling attenuates the dehydration-induced cell death and thereby promotes better adaptation of BiP-overexpressing lines to drought.

### The gradual dehydration of soybean leaves induces UPR/ERAD

The heatmap of UPR-specific genes revealed that progressive drought induces the UPR key transducer bZIP28 and UPR marker genes, such as SP1, bZIP60 and a subset of CNX and CRT (compare WTirr×WTψ_w_ = −2.0; Figure 5). The UPR induction caused by a gradual decline in the leaf ψ_w_ was further confirmed by a qRT-PCR of selected UPR marker genes (Figure 9). The activation of bZIP28- and bZIP60-supported branches in the UPR up-regulates ERAD genes as part of the quality control of the organelle [16], [61], [62]. The heatmap of ERAD marker genes at different levels of leaf ψ_w_ showed that these ERAD genes may be induced as a result of the decline of leaf ψ_w_, which supports the notion that UPR may be activated in soybean leaves in response to a gradual loss of water.

However, severe drought stress induced in soybean plants by withholding irrigation for seven days or in soybean seedlings by treating with high concentrations of PEG has been previously shown to repress the UPR [33], [34]. Under these conditions, severe osmotic stress down-regulates the expression of UPR marker genes, such as BiP, CNX and PDI. Contrasting results were also reported in previous studies which examined the BiP expression, as the sole UPR marker, in response to osmotic stress and drought. In fact, drought has been shown to reduce the BiP transcript levels in spinach, but a subset of BiP genes are up-regulated by osmotic stress, water deficit or ABA treatment in soybeans and tobacco [39], [40], [63]. The apparent contradiction in these results has been reconciled with the argument that BiP levels are controlled by the background status of plant cells in which the BiP basal levels and the cell secretory activity would signal the need for BiP positive or negative regulation under drought. As a UPR regulator, the cell may monitor the BiP levels as an indicator of the folding environment in the ER. More recently, genomic scale information on stress-induced changes has allowed for a more in-depth view of the scenario needed for reprogramming plant gene expression as a result of the interaction of the plant with the environment. In fact, the results of the present investigation in comparison with those regarding severe osmotic stress-induced changes in soybeans [34] clearly demonstrated that the activation or repression of UPR in response to drought conditions is totally dependent on the severity of the osmotic stress induced in the plant. Under slow osmotic stress induced by the gradual decline of leaf ψ_w_, UPR marker genes are up-regulated, and a rapid and severe osmotic stress represses UPR.

## Conclusions

The results of the present investigation provided several lines of evidence indicating that the underlying mechanism for BiP-mediated increases in water stress tolerance may be linked to a functional protective role against cellular dehydration and cell integrity as well as a regulatory role in stress-induced signaling pathways. In fact, at a similar leaf water status of moderate induced dehydration stress, BiP overexpression prevents drastic gene expression reprogramming, which may be explained by the ability of BiP to maintain cellular homeostasis. This interpretation was supported by directly comparing the size of drought-induced changes and the drought-induced transcriptomes of WT and 35S::BiP-4 lines at different leaf water statuses. In WT leaves, the ψ_w_ of −1.0 MPa was low enough to elicit a typical drought response as judged by the identity and number of differentially regulated genes; the 35S::BiP-4 stressed leaves required a decline in the ψ_w_ to −1.6 to sense the deleterious effects of dehydration and activate a shared repertory of drought responsive genes. Furthermore, BiP overexpression was associated with an inhibition of drought-induced PCD genes, which was narrowed down to ER stress and osmotic stress NRP-mediated cell death signaling. The delay and impairment of drought-induced senescence has been demonstrated to be linked to drought tolerance [58]. BiP overexpression also inhibited the progressive drought-mediated activation of UPR. BiPs from eukaryotic organisms as diverse as mammals, yeast and Arabidopsis have been shown to play a regulatory role in controlling the activation of UPR transducers. Soybean BiP is likely to share a similar conserved mechanism for the inhibition of UPR genes. Both the BiP functional role in protecting cellular proteins and structures and the BiP regulatory role in modulating stress-induced signaling pathways may be dependent on BiP binding to client proteins and may conceptually require its chaperone activity for function.

## Supporting Information

Figure S1 **MA plots of arrays as a function of log_2_ changes. M = log_2_fold change and A = average of intensity log.**
(TIF)

Table S1 **Gene-specific primers for qRT-PCR.**
(DOCX)

Table S2 **Number of up- and down-regulated genes in stressed WT and 35S::BiP-4 leaves compared to well-watered WT leaves.** Each functional category was defined by Gene Ontology.
(XLSX)

Table S3 **Number of up- and down-regulated genes in stressed 35S::BiP-4 leaves compared to well-watered 35S::BiP-4 leaves.** Each category was defined by Gene Ontology.
(XLSX)

Table S4 **Functional categorization of up-regulated genes in WT(−1.0)19d-WTIrrigated.**
(XLSX)

Table S5 **Functional categorization of down-regulated genes in WT(−1.0)19d-WTIrrigada.**
(XLSX)

Table S6 **Functional categorization of up-regulated genes in WT(−1.7)23d-WTIrrigated.**
(XLSX)

Table S7 **Functional categorization of down-regulated genes in WT(−1.7)23d-WTIrrigated.**
(XLSX)

Table S8 **Functional categorization of up-regulated genes in WT(−2.0) 25d-WTIrrigated.**
(XLSX)

Table S9 **Functional categorization of down-regulated genes in WT(−2.0)25d-WTIrrigated.**
(XLSX)

Table S10 **Functional categorization of up-regulated genes in (A) 35S::BiP-4(−1.0)21d-WTIrrigated and (B) 35S::BiP-4(−1.0)21d-35S::BiP-4Irrigated.**
(XLSX)

Table S11 **Functional categorization of down-regulated genes in (A) 35S::BiP-4 (−1.0)21d-WTIrrigated and (B) 35S::BiP-4 (−1.0)21d-35S::BiP-4Irrigated.**
(XLSX)

Table S12 **Functional categorization of up-regulated genes in (A) 35S::BiP-4(−1.6)25d-Wtirrigated and (B) 35S::BiP-4(−1.6)25d-35S::BiP-4irrigated.**
(XLSX)

Table S13 **Functional categorization of down-regulated genes in (A) 35S::BiP-4(−1.6)25d-Wtirrigated and (B) 35S::BiP-4(−1.6)25d-35S::BiP-4irrigated.**
(XLSX)

Table S14 **Functional categorization of (A) up-regulated genes and (B) down-regulated genes in 35S::BiP-4 compared to WT, under normal conditions.**
(XLSX)

Table S15 **Differential expression of NAC genes in WT(−1.0)19d-WTIrrigated, in WT(−1.7)23d-Wtirr and in WT(−2.0)25d-Wtirrigated.**
(XLSX)

Table S16 **(A) 35S::BiP-4(1,0)21d-Wtirr-specific up-regulated genes and (B) 35S::BiP-4(1,0)21d-35S::BiP-4irr-specific up-regulated genes in comparison with WT(−1,0)19d-WTIrrigated.**
(XLSX)

Table S17 **WT(−1,0)19d-WTIrrigated-specific up-regulated genes in comparison with (A) 35S::BiP-4(1,0)21d-Wtirr and (B) 35S::BiP-4(1,0)21d-35S::BiP-4irr.**
(XLSX)

Table S18 **(A) 35S::BiP-4 (−1,0)21d-WTIrri- and (B) 35S::BiP-4 (−1,0)21d-35S::BiP-4irr-specific down-regulated genes in comparison with WT(-1,0)19d-Wtirr.**
(XLSX)

Table S19 **WT(−1,0)19d-WTIrri-specific down-regulated genes in comparison with (A) 35S::BiP-4 (−1,0)21d-Wtirrigated and (B) 35S::BiP-4 (−1,0)21d-35S::BiP-4irrigated.**
(XLSX)

Table S20 **(A) 35S::BiP-4(−1,6)25d-WTIrri- and (B) 35S::BiP-4(−1,6)25d-35S::BiP-4Irri-specific up-regulated genes in comparison with WT(−1,7)23d–Wtirrigated.**
(XLSX)

Table S21 **WT(−1,7)23d-WTIrri-specific up-regulated genes in comparison with (A) 35S::BiP-4(−1,6)25d-Wtirrigated and (B) 35S::BiP-4(−1,6)25d-35S::BiP-4irrigated.**
(XLSX)

Table S22 **(A) 35S::BiP-4(−1,6)25d-WTIrri- and (B) 35S::BiP-4(−1,6)25d-35S::BiP-4Irri-specific down-regulated genes in comparison with WT(−1,7)23d-Wtirrigated.**
(XLSX)

Table S23 **WT(−1,7)23d–WTIrri-specific down-regulated genes in comparison with (A) 35S::BiP-4(−1,6)25d-Wtirrigated and (B) 35S::BiP-4(−1,6)25d-35S::BiP-4irrigated.**
(XLSX)
