# Supplementary figures and images for: The Molecular Chaperone Binding Protein BiP Prevents Leaf Dehydration-Induced Cellular Homeostasis Disruption
Source: PLoS One. 2014 Jan 29;9(1):e86661. doi: 10.1371/journal.pone.0086661 (PMC3906070; doi:10.1371/journal.pone.0086661)

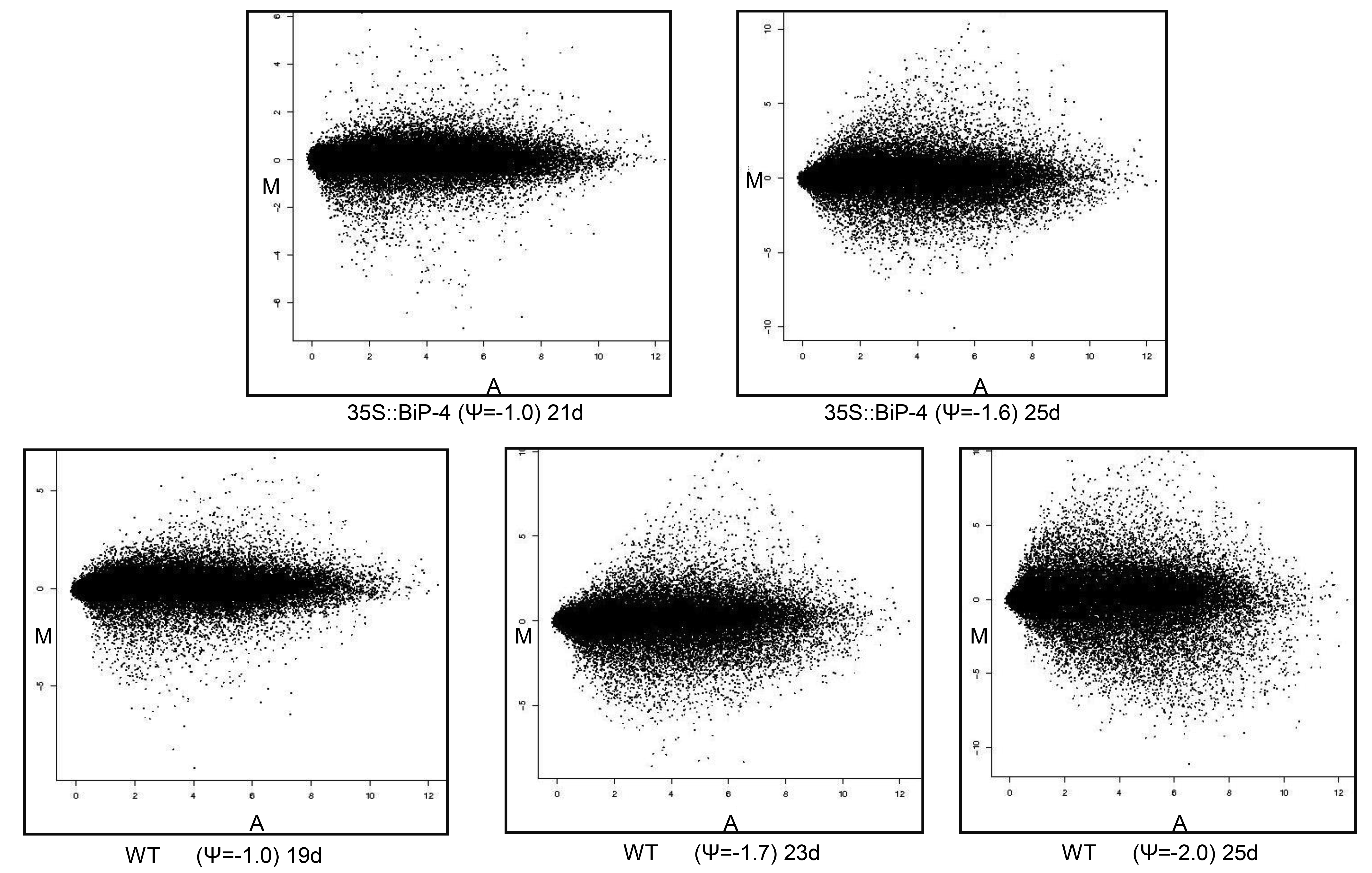

Supplement: Figure S1 — MA plots of arrays as a function of log2 changes. M = log2fold change and A = average of intensity log. (TIF) [file pone.0086661.s001.tif]
