# Supplementary material for: The Molecular Chaperone Binding Protein BiP Prevents Leaf Dehydration-Induced Cellular Homeostasis Disruption
Source: PLoS One. 2014 Jan 29;9(1):e86661. doi: 10.1371/journal.pone.0086661 (PMC3906070; doi:10.1371/journal.pone.0086661)
Supplement: Table S1 — Gene-specific primers for qRT-PCR. (DOCX) [file pone.0086661.s002.docx]

**Table S1. Gene-specific primers for qRT-PCR**

| **Primers** | **Sequence (5’ – 3’)** | **Gene** |
| --- | --- | --- |
| BiP trans Fw | ATCTGGAGGAGCCCTAGGCGGTGG | BiPD - nos |
| BiP trans Rv | CATCGCAAGACCGGCAACAGGAT |  |
| Helic Fw | TAACCCTAGCCCCTTCGCCT | HELICASE |
| Helic Rv | GCCTTGTCGTCTTCCTCCTCG | AI736067 |
| Nac6 Fw | CCAACAAAAGCACTTGTGGCA | NAC6 |
| Nac6 Rv | GGACTATTCAACTGAGCCCAAAAG | Glyma11g10230 |
| Calnexin | TGATGGGGAGGAGAAGAAAAAGGC | CNX |
| Calnexin | ATCTGGAGGAGCCCCAGGCGGTGG | AW508066 |
| BiPD Fw | ATCTGGAGGAGCCCCAGGCGGTGG | BiPD |
| BiPD Rv | CTTGAAGAAGCTTCGTCGTAAAACTAAG | AF031241 |
| GST Fw | CGGTTCTCATCCACAATGGCAAAC | GST |
| GST Rv | CAGCCCAGAATCTAGCCTGAGC | Glyma15g40190 |
| NAC3 Fw | GAATGCAGCAATGGGTCATCA | Nac3 |
| NAC3 Rv | ATCCTGCTGGTGCATTGTTCTG | AY974351 |
| NRP Fw | GGCACAAAGACTGGTGCTGAGA | NRP-A |
| NRP Rv | CTCTGTATCGTGGAGGCAGACC | AJ875407 |
| NRich Fw | TACAGGCATCCAATTTGGCGAACC | NRP-B |
| NRich Rv | TGACTTGAAAGAGTTGATCTCACCCC | AI973541 |
| SMP Fw | GCCGAACTGAGGAAAAGACGAACC | SMP |
| SMP Rv | CTTGGGCTGTTTGTTGGGTCTTC | AW397921 |
| NAC1 Fw | GGACTACCCAATAGCCCAAATCA | NAC1 |
| NAC1 Rv | GACCCAAGTAATCCATTTCCAAAAG | AY974349 |
| Glyma02g19754Fwd | GCTTCCGATGAACCCATGTC | Glyma02g1975 |
| Glyma02g19754Rvs | TTCCTCTCCCTTGACCTCACA |  |
| Glyma03g27865Fwd | CAGTCGTCAGGGCAATGCT | Glyma03g27865Fwd |
| Glyma03g27865Rvs | CATCAATCTTCACCATCTTATCATTCC |  |
| Glyma19g30681Fwd | TCACCAATGACTGCGTCAATG | Glyma19g30681Rvs |
| Glyma19g30681Rvs | GAGAAGTCGCCAAACACGAGAT |  |
| VPE Fw | AACCCAAGGCCTGGAGTCAT | VPE |
| VPE Rv | TCGCCGGTGTAATCCTTTG | Glyma14g10620 |
